# Precision identification and targeted therapy for neutrophilic asthma: from molecular mechanisms to clinical translation

**DOI:** 10.3389/fimmu.2026.1878339

**Published:** 2026-07-08

**Authors:** Mengmeng Sun, Yan Xu, Guihua Song, Bingxue Zhang, MingHao Peng, SuPing Yu, GuiChun Zhang

**Affiliations:** 1 Department of Pediatrics, First Affiliated Hospital, Henan University of Chinese Medicine, Zhengzhou, Henan, China; 2 School of Pediatrics, Henan University of Chinese Medicine, Zhengzhou, Henan, China

**Keywords:** glucocorticoid resistance, microbiome, neutrophil extracellular traps, neutrophilic asthma, NLRP3 inflammasome, precision medicine, Th17/IL-17 axis

## Abstract

Neutrophilic asthma represents a distinct inflammatory phenotype characterized by sputum neutrophilia (≥61% neutrophils), glucocorticoid resistance, and more severe disease course compared to eosinophilic asthma. This review comprehensively examines the molecular mechanisms underlying neutrophilic asthma pathogenesis, focusing on the Th17/IL-17 axis, neutrophil extracellular traps (NETs), and NLRP3 inflammasome activation. We present a precision identification framework integrating molecular endotypes with clinical phenotypes and biomarker profiles to guide therapeutic decisions. Unlike eosinophilic asthma, neutrophilic asthma demonstrates intrinsic resistance to glucocorticoids due to impaired neutrophil apoptosis and persistent activation of pro-inflammatory pathways. Emerging therapeutic approaches targeting IL-17, NET formation, and inflammasome components show promise, with several agents in clinical development. The microbiome-neutrophil axis represents a novel therapeutic target, with evidence suggesting that airway dysbiosis perpetuates neutrophilic inflammation through pattern recognition receptor activation. This review provides a comprehensive framework for understanding neutrophilic asthma pathogenesis and outlines precision medicine approaches for this difficult-to-treat asthma phenotype.

## 1 Introduction

Asthma affects over 300 million people worldwide and is traditionally classified as a Th2-mediated eosinophilic disease (1–3). However, approximately 10-20% of asthma patients exhibit neutrophilic inflammation, characterized by elevated sputum neutrophils (≥61%) and distinct pathophysiological mechanisms (4, 5). Neutrophilic asthma is associated with more severe disease, accelerated lung function decline, and poor response to conventional glucocorticoid therapy, representing a significant unmet clinical need (4, 6–8).

The recognition of neutrophilic asthma as a distinct phenotype emerged from cluster analyses demonstrating differential inflammatory patterns and therapeutic responses (4, 5). Unlike eosinophilic asthma, which responds well to inhaled corticosteroids (ICS) and biologics targeting IL-5 or IgE, neutrophilic asthma exhibits inherent glucocorticoid resistance and lacks approved targeted therapies (6, 9). This therapeutic gap underscores the need for improved understanding of disease mechanisms and development of novel treatment strategies.

Recent advances in immunology and molecular biology have elucidated key pathways driving neutrophilic inflammation in asthma. The Th17/IL-17 axis plays a central role in neutrophil recruitment and activation (10, 11), while neutrophil extracellular traps (NETs) contribute to airway damage and hyperresponsiveness (12, 13). The NLRP3 inflammasome amplifies inflammation through IL-1β and IL-18 release, creating a self-perpetuating inflammatory cycle (14, 15). Additionally, emerging evidence implicates airway microbiome dysbiosis in neutrophilic asthma pathogenesis, suggesting novel therapeutic targets (16, 17).

This review synthesizes current knowledge of neutrophilic asthma molecular mechanisms, presents a framework for precision identification, and evaluates emerging therapeutic approaches. We aim to provide clinicians and researchers with a comprehensive understanding of this challenging asthma phenotype and practical guidance for implementing precision medicine strategies.Key distinguishing features between neutrophilic and eosinophilic asthma across clinical, inflammatory, and therapeutic dimensions are summarized in **Table 1**.

**Table 1:** Comparison of eosinophilic and neutrophilic asthma phenotypes.

Characteristic	Eosinophilic asthma	Neutrophilic asthma
Sputum Differential	Eosinophils ≥3%	Neutrophils ≥61%
Typical Age of Onset	Childhood or adult	Adult
Atopy	Common	Less common
Disease Severity	Mild to severe	Often moderate to severe
Glucocorticoid Response	Good	Poor/Variable
FeNO Level	Elevated	Normal/Low
Key Cytokines	IL-4, IL-5, IL-13	IL-17, IL-8, IL-1β
Approved Biologics	Anti-IgE, Anti-IL-5, Anti-IL-4Rα	None specific (azithromycin off-label)
Primary Cells	Th2 cells, Eosinophils	Th17 cells, Neutrophils
Comorbidities	Allergic rhinitis, Atopic dermatitis	Chronic rhinosinusitis, Bronchiectasis

## 2 Molecular mechanisms of neutrophilic asthma

### 2.1 Th17/IL-17 axis and neutrophil recruitment

The Th17/IL-17 axis represents the cornerstone of neutrophilic asthma pathogenesis (10, 11). Th17 cells, a distinct CD4+ T helper subset, produce IL-17A, IL-17F, IL-21, and IL-22, which orchestrate neutrophil recruitment and activation (11, 18). IL-17A and IL-17F signal through the IL-17RA/IL-17RC heterodimeric receptor, activating downstream pathways including NF-κB, C/EBPβ, and MAPK signaling (10).

#### 2.1.1 Neutrophil chemotaxis mechanisms

IL-17 induces epithelial cells and airway smooth muscle cells to produce CXCL1, CXCL2, CXCL5, and CXCL8 (IL-8), potent neutrophil chemoattractants. These chemokines create a chemotactic gradient that drives neutrophil migration from the circulation into the airway lumen. Additionally, IL-17 upregulates G-CSF, promoting neutrophil production and release from bone marrow.

Studies demonstrate elevated IL-17A levels in sputum, bronchoalveolar lavage fluid, and serum of neutrophilic asthma patients, correlating with disease severity and neutrophil counts (11, 18). IL-17A+ CD4+ T cells are increased in bronchial biopsies of severe asthma patients with neutrophilic inflammation (10, 11). Genetic studies support the causal role of IL-17, with polymorphisms in IL-17 pathway genes associated with asthma severity and neutrophilic inflammation (11, 19).

#### 2.1.2 Th17 cell differentiation and plasticity

Th17 differentiation requires TGF-β, IL-6, and IL-1β in mice, with IL-1β and IL-23 being critical for human Th17 development (10, 20). The transcription factors RORγt and STAT3 drive Th17 lineage commitment. Importantly, Th17 cells demonstrate remarkable plasticity, capable of converting to Th1-like cells producing IFN-γ, acquiring regulatory functions, or even co-producing Th2 cytokines such as IL-4 under certain conditions (10, 19, 21). This plasticity has implications for disease progression and therapeutic targeting.

#### 2.1.3 Antigen-driven Th17 expansion

Beyond canonical differentiation from naïve T cells, Th17 responses are amplified through antigen-specific mechanisms (10, 18). Dendritic cells presenting bacterial or viral antigens in the context of IL-6 and IL-23 drive robust Th17 expansion (19). In neutrophilic asthma, chronic airway colonization with pathogenic bacteria such as Haemophilus influenzae and Moraxella catarrhalis provides persistent antigenic stimulation, sustaining Th17 responses (22, 23). Cross-presentation of fungal antigens from airway colonization with Aspergillus species similarly augments Th17 activity (19, 20). This antigen-driven expansion creates a feed-forward loop where tissue damage releases damage-associated molecular patterns (DAMPs), further activating dendritic cells and amplifying Th17-mediated neutrophil recruitment (10, 19).

### 2.2 Neutrophil extracellular traps in airway inflammation

Neutrophil extracellular traps (NETs) are web-like structures composed of chromatin DNA, histones, and granule proteins released by activated neutrophils (12, 24). While NETs serve antimicrobial functions, excessive or dysregulated NET formation contributes to airway pathology in neutrophilic asthma (13, 25).

#### 2.2.1 Mechanisms of NET formation

NET release occurs through two primary pathways: (1) suicidal NETosis, a lytic cell death process requiring NADPH oxidase activation, histone citrullination by PAD4, and nuclear envelope rupture; and (2) vital NETosis, a rapid release mechanism preserving neutrophil viability (12, 24). In neutrophilic asthma, both pathways are activated by inflammatory stimuli including IL-8, LPS, and immune complexes (13, 26).

#### 2.2.2 Pathological consequences of NETs

NET components directly damage airway epithelium and contribute to airway hyperresponsiveness (24, 26). Histones exhibit cytotoxic effects on epithelial cells, while neutrophil elastase and matrix metalloproteinases degrade extracellular matrix and promote mucus hypersecretion (13, 24). NETs also impair macrophage-mediated efferocytosis, prolonging inflammation. Studies demonstrate increased NET markers (cell-free DNA, citrullinated histone H3) in sputum of severe asthma patients, correlating with neutrophil counts and disease severity (13, 26, 27).

#### 2.2.3 NET-mediated inflammasome activation

NETs serve as endogenous danger signals, activating the NLRP3 inflammasome through multiple mechanisms (14, 28). NET-associated DNA activates AIM2 inflammasomes in macrophages, while extracellular histones trigger TLR4-NLRP3 signaling (14, 15). This NET-inflammasome axis amplifies IL-1β and IL-18 production, further driving neutrophil recruitment and creating a self-perpetuating inflammatory cycle (15, 28). Therapeutic targeting of NET formation or clearance may interrupt this amplification loop (24, 26).

### 2.3 NLRP3 inflammasome activation

The NLRP3 inflammasome, a multiprotein complex assembled in response to diverse danger signals, represents a critical amplifier of neutrophilic inflammation (14, 28). Inflammasome activation results in caspase-1-mediated cleavage of pro-IL-1β and pro-IL-18 into their active forms, driving inflammatory cascades (15, 28).

#### 2.3.1 Activation mechanisms

NLRP3 inflammasome assembly requires two signals: (1) priming through NF-κB activation (e.g., by LPS or IL-17) upregulating NLRP3 and pro-IL-1β expression; and (2) activation by danger-associated molecular patterns (DAMPs) including ATP, crystalline particles, and ROS (14, 15). In neutrophilic asthma, airway neutrophils release multiple NLRP3 activators including ATP, mitochondrial DNA, and NETs, creating autocrine and paracrine activation loops (15, 29).

#### 2.3.2 Clinical relevance

Elevated NLRP3, caspase-1, IL-1β, and IL-18 levels are observed in sputum and bronchial biopsies of neutrophilic asthma patients (15, 29). Genetic studies link NLRP3 polymorphisms to asthma severity (15). NLRP3 inhibition reduces neutrophilic inflammation in preclinical asthma models, supporting therapeutic targeting (15, 28).

#### 2.2.3 Crosstalk with IL-17 pathway

IL-17 synergizes with NLRP3 inflammasome signaling (10, 15). IL-17 enhances pro-IL-1β expression through NF-κB, priming inflammasome activation (15). Conversely, IL-1β promotes Th17 differentiation, creating an IL-17/IL-1β amplification loop (20, 28). This crosstalk suggests combined IL-17 and NLRP3 targeting may provide additive therapeutic benefit (15, 30).

### 2.4 Mechanisms of glucocorticoid resistance

**Figure 1:**
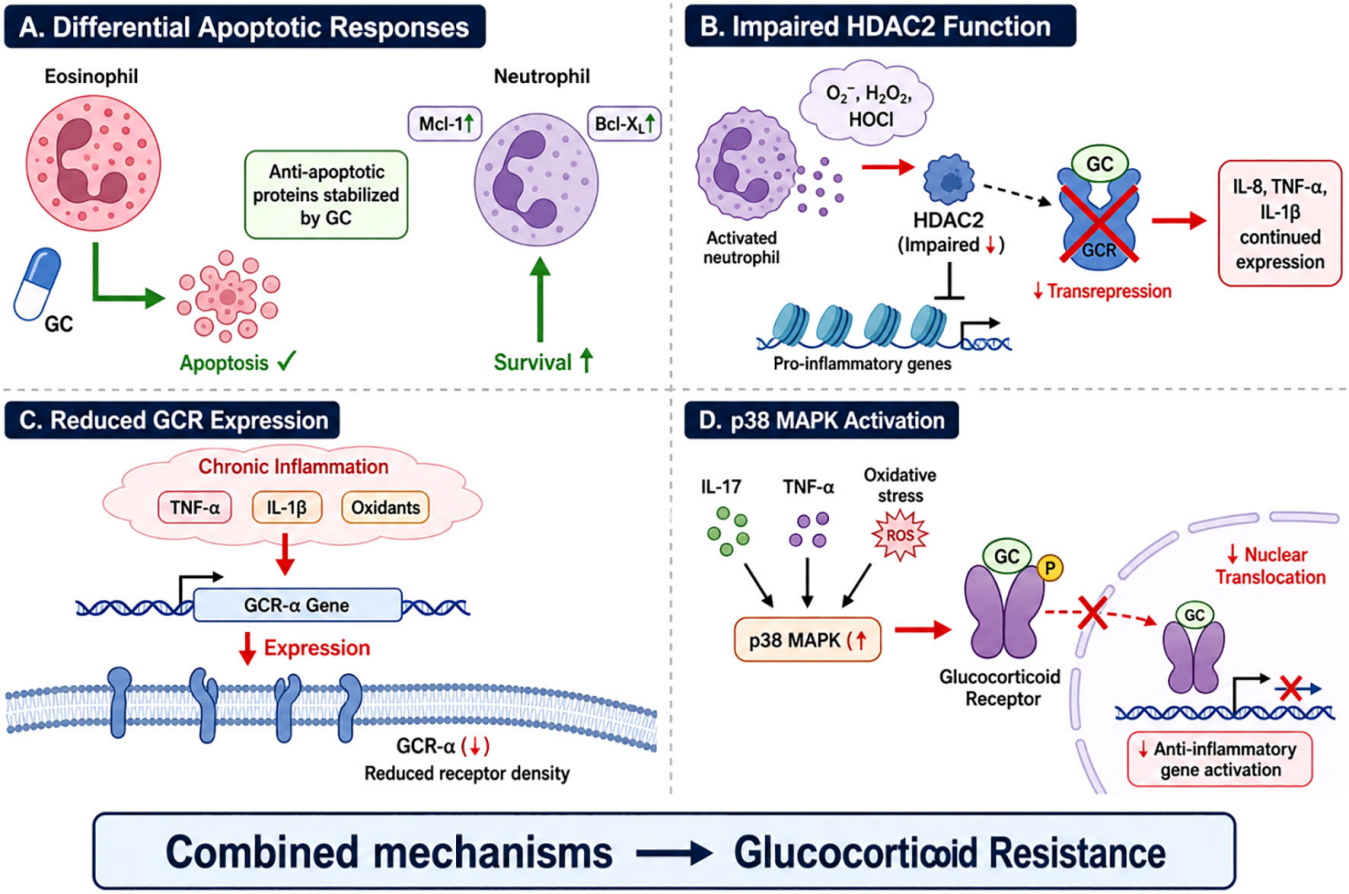
Mechanisms of glucocorticoid resistance. Cellular and molecular mechanisms underlying glucocorticoid resistance in neutrophilic asthma. **(A)** Differential apoptotic responses of eosinophils and neutrophils to glucocorticoids. Eosinophils undergo GC-induced apoptosis, while neutrophils express anti-apoptotic proteins (Mcl-1, Bcl-XL) that are further stabilized by GCs, promoting survival. **(B)** Impaired HDAC2 function due to oxidative modification by neutrophil-derived reactive oxygen species (O_2_^−^, H_2_O_2_, HOCl). Damaged HDAC2 cannot be recruited by GC-GCR complex, resulting in diminished transrepression of pro-inflammatory genes (IL-8, TNF-α, IL-1β). **(C)** Reduced glucocorticoid receptor (GCR-α) expression from chronic inflammatory cytokine exposure (TNF-α, IL-1β) and oxidants, decreasing receptor density at cell membrane. **(D)** p38 MAPK activation by IL-17 and cellular stress leads to GCR phosphorylation, impairing nuclear translocation and reducing anti-inflammatory gene activation.

Glucocorticoids (GCs) are the cornerstone of asthma management, yet neutrophilic asthma demonstrates intrinsic resistance to GC therapy (6, 9). Understanding the molecular basis of GC resistance is essential for developing alternative therapeutic strategies (6, 31) (**Figure 1**).

#### 2.4.1 Neutrophil-specific GC responses

Unlike eosinophils, which undergo GC-induced apoptosis, neutrophils are resistant to GC-mediated cell death (6, 32). This differential response reflects fundamental differences in apoptotic machinery between cell types (32). Neutrophils express high levels of anti-apoptotic proteins including Mcl-1 and Bcl-XL, which are further stabilized by inflammatory cytokines (IL-8, G-CSF, TNF-α) present in the neutrophilic airway (6, 32). Glucocorticoids paradoxically enhance Mcl-1 expression in neutrophils, prolonging their survival rather than inducing apoptosis (9, 32).

#### 2.4.2 Impaired histone deacetylase activity

Glucocorticoid efficacy depends on recruitment of histone deacetylases (HDACs) to pro-inflammatory gene promoters (9, 33). In neutrophilic asthma, oxidative stress from activated neutrophils (superoxide anion, hydrogen peroxide, hypochlorous acid) causes oxidative modification of HDAC2, impairing its function (31, 33). Reduced HDAC2 activity diminishes GC-mediated transrepression of pro-inflammatory genes, including those encoding IL-8, TNF-α, and IL-1β (9, 33). This mechanism is particularly relevant in severe asthma with persistent neutrophilic inflammation (31, 33).

#### 2.4.3 Reduced glucocorticoid receptor expression

Chronic exposure to inflammatory cytokines (TNF-α, IL-1β) and oxidants can downregulate glucocorticoid receptor (GCR) expression, particularly the GCR-α isoform required for anti-inflammatory signaling (6, 33). Neutrophilic inflammation creates a microenvironment that perpetuates reduced GCR expression and impaired GC responsiveness (6, 31).

#### 2.4.4 P38 MAPK activation

The p38 MAPK pathway, activated by cellular stress and inflammatory signals, phosphorylates GCR and reduces its nuclear translocation efficiency (6, 34). In neutrophilic asthma, persistent activation of p38 MAPK by IL-17, TNF-α, and oxidative stress contributes to GC insensitivity (33, 34). Inhibition of p38 MAPK restores GC sensitivity *in vitro*, suggesting a potential combination therapy approach (31, 34).

### 2.5 Microbiome dysbiosis and neutrophilic inflammation

Emerging evidence implicates airway and gut microbiome dysbiosis in neutrophilic asthma pathogenesis (16, 17). The microbiome-neutrophil axis represents a novel therapeutic target with implications for disease prevention and treatment (22, 35).

#### 2.5.1 Airway dysbiosis in neutrophilic asthma

Studies utilizing 16S rRNA sequencing demonstrate altered airway microbiome composition in neutrophilic asthma patients (22, 36). Characteristic patterns include enrichment with Proteobacteria (particularly Haemophilus, Moraxella, and Neisseria species) and reduced microbial diversity (23, 36). This dysbiotic profile correlates with sputum neutrophil counts, IL-17 levels, and disease severity (22, 23). Mechanistic studies suggest that pathogenic bacteria drive neutrophilic inflammation through pattern recognition receptor (PRR) activation, particularly TLR4 and NOD2 signaling (16, 22).

#### 2.5.2 Gut-lung axis

The gut microbiome influences lung immunity through systemic immune modulation and metabolite production (16, 17). Short-chain fatty acids (SCFAs), particularly butyrate and propionate, produced by gut bacterial fermentation of dietary fiber, exert anti-inflammatory effects through G-protein coupled receptor (GPCR) signaling and histone deacetylase inhibition (17, 35). Reduced SCFA production from gut dysbiosis may contribute to neutrophilic inflammation (35, 37). Epidemiological studies link early-life antibiotic exposure, which disrupts gut microbiome, to increased asthma risk, particularly non-atopic and severe phenotypes (35, 37).

#### 2.5.3 Mechanisms linking dysbiosis to neutrophilic inflammation

Dysbiotic airway microbiomes perpetuate neutrophilic inflammation through multiple mechanisms: (1) Direct activation of epithelial cells by bacterial products (LPS, flagellin) inducing IL-8 and G-CSF production (16, 22); (2) Antigen presentation driving Th17 differentiation and expansion (10, 23); (3) Biofilm formation providing persistent antigenic stimulation and resistance to immune clearance (38); (4) Metabolite production (e.g., phenazines from Pseudomonas) directly activating neutrophils and prolonging survival (16). The resultant chronic neutrophilic infiltration damages airway epithelium, creating niches for further bacterial colonization—a pathogenic cycle perpetuating both dysbiosis and inflammation (22, 38).

#### 2.5.4 Therapeutic implications

Microbiome-targeted interventions show promise in preclinical models (16, 35). Probiotic administration reduces neutrophilic inflammation in murine asthma models, while fecal microbiome transplantation (FMT) ameliorates disease severity (35). Antibiotic treatment in patients with chronic airway infection reduces sputum neutrophils, though the non-specific nature of antibiotics limits long-term utility (23, 38). Development of precision microbiome interventions, including targeted bacteriophage therapy and rational probiotic formulations, represents an emerging therapeutic frontier (16, 35).

### 2.6 Integrated pathogenic network

**Figure 2:**
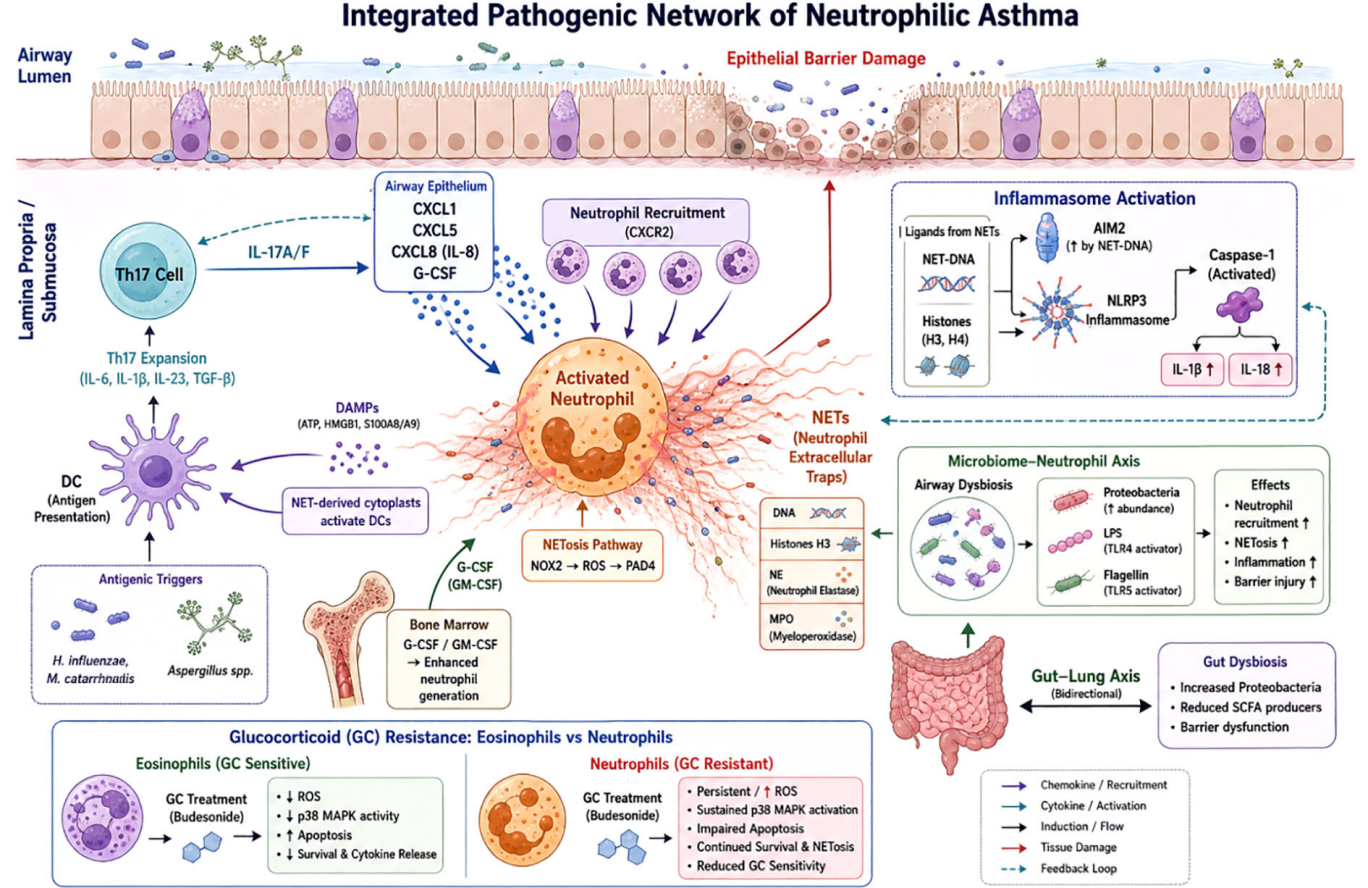
Integrated pathogenic network of neutrophilic asthma. Comprehensive schematic of the interconnected molecular mechanisms driving neutrophilic asthma. The central network illustrates Th17/IL-17 axis signaling: Th17 cells produce IL-17A/F, inducing epithelial cell production of CXCL chemokines (CXCL1, CXCL5, CXCL8) and G-CSF, driving neutrophil recruitment from bone marrow. Antigen-driven amplification: dendritic cells presenting bacterial/fungal antigens (Haemophilus influenzae, Moraxella catarrhalis, Aspergillus) in the context of IL-6/IL-23 expand Th17 responses. Neutrophil extracellular traps (NETs): activated neutrophils release chromatin DNA, histones, neutrophil elastase (NE), and myeloperoxidase (MPO) through suicidal and vital NETosis pathways. NET-associated DNA activates AIM2 inflammasome, while extracellular histones trigger TLR4-NLRP3 signaling, producing IL-1β and IL-18. Microbiome-neutrophil axis: airway dysbiosis with Proteobacteria enrichment drives inflammation through LPS/TLR4 activation; gut microbiome dysbiosis reduces short-chain fatty acid (SCFA) production, diminishing anti-inflammatory signaling via GPR43/109A. IL-1β feeds back to promote Th17 differentiation, creating self-reinforcing inflammatory loops. Dashed arrows indicate feedback and amplification pathways.

The molecular mechanisms of neutrophilic asthma form an interconnected network where Th17/IL-17 signaling, NET formation, NLRP3 inflammasome activation, and microbiome dysbiosis create self-reinforcing inflammatory loops. IL-17 drives neutrophil recruitment and primes NLRP3 inflammasome expression. Recruited neutrophils release NETs and DAMPs that activate inflammasomes, producing IL-1β that further promotes Th17 differentiation. Dysbiotic airway microbiomes provide persistent antigenic stimulation driving Th17 expansion while bacterial products directly activate neutrophils and epithelial cells. Glucocorticoid resistance perpetuates this cycle by failing to resolve neutrophilic inflammation. Understanding this integrated pathogenic network informs therapeutic strategies targeting multiple nodes simultaneously (**Figure 2**).

### 3 Precision identification framework for neutrophilic asthma

Accurate identification of neutrophilic asthma is essential for appropriate therapeutic intervention (39, 40). This section presents a precision identification framework integrating molecular endotypes, biomarker profiles, and clinical phenotypes to guide diagnosis and treatment selection (39, 41).

### 3.1 Molecular endotypes

Neutrophilic asthma encompasses multiple molecular endotypes defined by distinct pathogenic mechanisms:

#### 3.1.1 IL-17-dominant endotype

Characterized by elevated IL-17A, IL-17F, and Th17-associated cytokines. Patients exhibit high sputum neutrophils, increased CXCL1/CXCL8 levels, and evidence of Th17 activation (IL-17+ CD4+ T cells in bronchial biopsies). This endotype may respond preferentially to IL-17 pathway inhibitors.

#### 3.1.2 Inflammasome-activated endotype

Features elevated IL-1β, IL-18, and NLRP3 inflammasome markers. Patients may have comorbidities associated with inflammasome activation (obesity, metabolic syndrome). IL-1β inhibition represents a rational therapeutic approach for this endotype.

#### 3.1.3 NET-associated endotype

Defined by elevated NET markers (cell-free DNA, citrullinated histone H3, neutrophil elastase-DNA complexes) in sputum or BAL. This endotype may benefit from therapies targeting NET formation (PAD4 inhibitors, DNase) or enhancing NET clearance.

#### 3.1.4 Infection-associated endotype

Occurs in the context of chronic bacterial colonization or recurrent infections. Airway culture identifies pathogenic bacteria (Haemophilus influenzae, Moraxella catarrhalis, Pseudomonas aeruginosa). Antibiotic therapy or microbiome-targeted interventions may be beneficial.The defining biomarkers, pathogenic mechanisms, therapeutic targets, and investigational agents for each endotype are summarized in **Table 2**.

**Table 2:** Molecular endotypes and targeted therapies in neutrophilic asthma.

Endotype	Key biomarkers	Pathogenic mechanism	Therapeutic targets	Investigational agents
IL-17-Dominant	↑ IL-17A, ↑ IL-17F, ↑ Th17 cells, ↑ CXCL1/8	Th17-driven neutrophil recruitment	IL-17A, IL-17RA, IL-23	Secukinumab, Ixekizumab, Risankizumab
Inflammasome-Activated	↑ IL-1β, ↑ IL-18, ↑ NLRP3, ↑ hsCRP	NLRP3 inflammasome amplification	NLRP3, IL-1β, IL-1R	MCC950, Canakinumab, Anakinra
NET-Associated	↑ cfDNA, ↑ CitH3, ↑ NE-DNA complexes, ↑ PAD4	NET-mediated tissue damage	PAD4, NET clearance	PAD4 inhibitors, DNase
Infection-Associated	Positive culture, ↑ bacterial load, ↓ diversity	Chronic bacterial colonization	Bacteria, Biofilm	Targeted antibiotics, Macrolides

cfDNA, cell-free DNA; CitH3, citrullinated histone H3; CXCL, C-X-C motif chemokine ligand; hsCRP, high-sensitivity C-reactive protein; IL, interleukin; NE, neutrophil elastase; NET, neutrophil extracellular trap; PAD4, peptidylarginine deiminase 4; Th17, T helper 17.

↑ indicates elevated levels; ↓ indicates decreased levels.

### 3.2 Biomarker profiles

#### 3.2.1 Sputum cell counts

Induced sputum cytology remains the gold standard for identifying neutrophilic asthma (≥61% neutrophils) (1, 4). Limitations include technical expertise requirements, sample adequacy concerns, and variability (4, 42). Standardized protocols and quality control measures improve reliability (1).

#### 3.2.2 Peripheral blood biomarkers

Blood neutrophil counts correlate moderately with sputum neutrophils but lack sufficient sensitivity and specificity for clinical use (7, 27). Emerging blood biomarkers include:

- Serum IL-17A and IL-8: Elevated in neutrophilic asthma but overlap with other inflammatory conditions (7, 27)
- Serum CXCL1 and CXCL5: Potential biomarkers requiring validation (7, 27)
- Blood neutrophil gene signatures: Transcriptomic profiles showing promise for endotype classification (7, 31)

#### 3.2.3 Exhaled breath analysis

Exhaled nitric oxide (FeNO) is typically low in neutrophilic asthma, helping differentiate from eosinophilic asthma (3, 41). Exhaled breath volatile organic compounds (VOCs) are under investigation as non-invasive biomarkers for inflammatory phenotypes (41).

#### 3.2.4 Imaging biomarkers

CT imaging may reveal airway wall thickening, mucus plugging, and bronchiectasis more common in neutrophilic asthma. PET-CT with FDG uptake correlates with airway inflammation but is not routinely used clinically.

#### 3.2.5 Composite biomarker panels

Integration of multiple biomarkers improves diagnostic accuracy (27, 41). Proposed panels combine sputum cytology, FeNO, serum cytokines (IL-17, IL-8), and clinical characteristics (7, 27). Machine learning approaches applied to multi-omics data (transcriptomics, proteomics, metabolomics) show promise for precision endotyping (39, 41).

### 3.3 Clinical phenotypes

Several clinical phenotypes associate with neutrophilic inflammation (7, 8):

#### 3.3.1 Severe asthma

Neutrophilic inflammation is overrepresented in severe asthma, particularly in patients with frequent exacerbations and fixed airflow limitation (4, 7). These patients often demonstrate GC resistance and require targeted therapies (6, 40).

#### 3.3.2 Late-onset non-atopic asthma

Patients with adult-onset asthma without atopic features frequently exhibit neutrophilic inflammation (5, 43). This phenotype may be triggered by environmental exposures, chronic infection, or obesity-related inflammation (8, 43).

#### 3.3.3 Obesity-associated asthma

Obesity creates a pro-inflammatory state with elevated IL-6, IL-1β, and systemic inflammation (8, 44). Obese asthma patients show increased neutrophilic inflammation relative to lean asthma patients, potentially explaining differential therapeutic responses (8, 44).

#### 3.3.4 Smoking-related asthma

Current or former smokers demonstrate increased sputum neutrophils and GC resistance (6, 33). Cigarette smoke exposure impairs HDAC function and activates neutrophil-inflammatory pathways (6, 33).

#### 3.3.5 Chronic airway infection

Patients with chronic bacterial colonization or bronchiectasis exhibit persistent neutrophilic inflammation (22, 38). This phenotype overlaps with bronchiectasis and requires comprehensive airway microbiology assessment (23, 38).

### 3.4 Integrated Precision Identification Algorithm

A stepwise approach to precision identification integrates clinical assessment, biomarker profiling, and molecular endotyping:

Step 1: Clinical Assessment

- Evaluate symptom pattern, exacerbation frequency, and treatment response
- Identify comorbidities (obesity, chronic rhinosinusitis, GERD)
- Assess risk factors (smoking history, occupational exposures, infection history)

Step 2: Baseline Biomarker Testing

- Induced sputum cytology (if available)
- FeNO measurement
- Blood eosinophil count, total IgE
- Serum IL-17A, IL-8 (if available)

Step 3: Endotype Determination

- If sputum neutrophils ≥61%: Consider molecular endotyping
- IL-17 pathway analysis (serum IL-17, Th17 cell frequency)
- Inflammasome markers (serum IL-1β, IL-18)
- NET markers (sputum cell-free DNA, citrullinated histone H3)
- Airway microbiology (culture, molecular detection)

Step 4: Therapeutic Decision Integration

**Figure 3:**
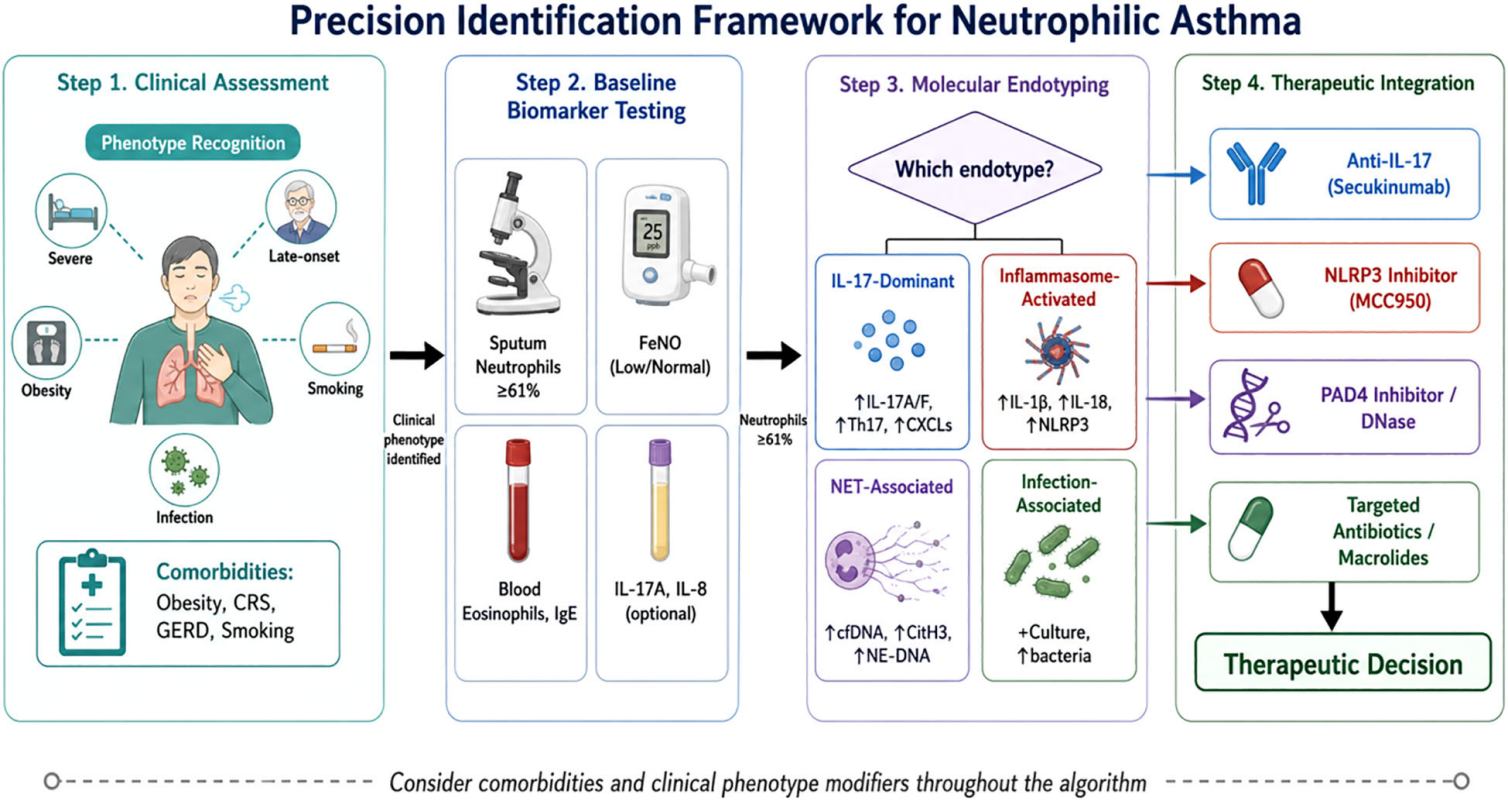
Precision identification framework for neutrophilic asthma. Stepwise algorithm integrating clinical phenotypes, biomarker profiles, and molecular endotypes for precision diagnosis. Step 1: Clinical assessment identifies phenotypes associated with neutrophilic inflammation—severe asthma, late-onset non-atopic, obesity-associated, smoking-related, and infection-associated patterns. Comorbidity evaluation includes obesity, chronic rhinosinusitis, GERD, and smoking history. Step 2: Baseline biomarker testing with induced sputum cytology (≥61% neutrophils as gold standard), FeNO (typically low), blood eosinophils, IgE, and optional serum cytokines (IL-17A, IL-8). Step 3: Molecular endotyping into four categories: IL-17-dominant (↑IL-17A/F, ↑Th17, ↑CXCLs), inflammasome-activated (↑IL-1β, ↑IL-18, ↑NLRP3), NET-associated (↑cfDNA, ↑CitH3, ↑NE-DNA complexes), and infection-associated (positive culture, bacterial colonization). Step 4: Therapeutic integration matching endotypes to targeted treatments—anti-IL-17 for IL-17-dominant, NLRP3/IL-1β inhibitors for inflammasome-activated, PAD4 inhibitors/DNase for NET-associated, and targeted antibiotics for infection-associated endotypes.

- Match endotype to targeted therapies (see Section 4)
- Consider clinical phenotype modifiers (obesity, smoking, infection)
- Evaluate comorbidities requiring concurrent management (**Figure 3**)

## 4 Therapeutic strategies

### 4.1 Clinical implications and personalized treatment approaches

**Figure 4:**
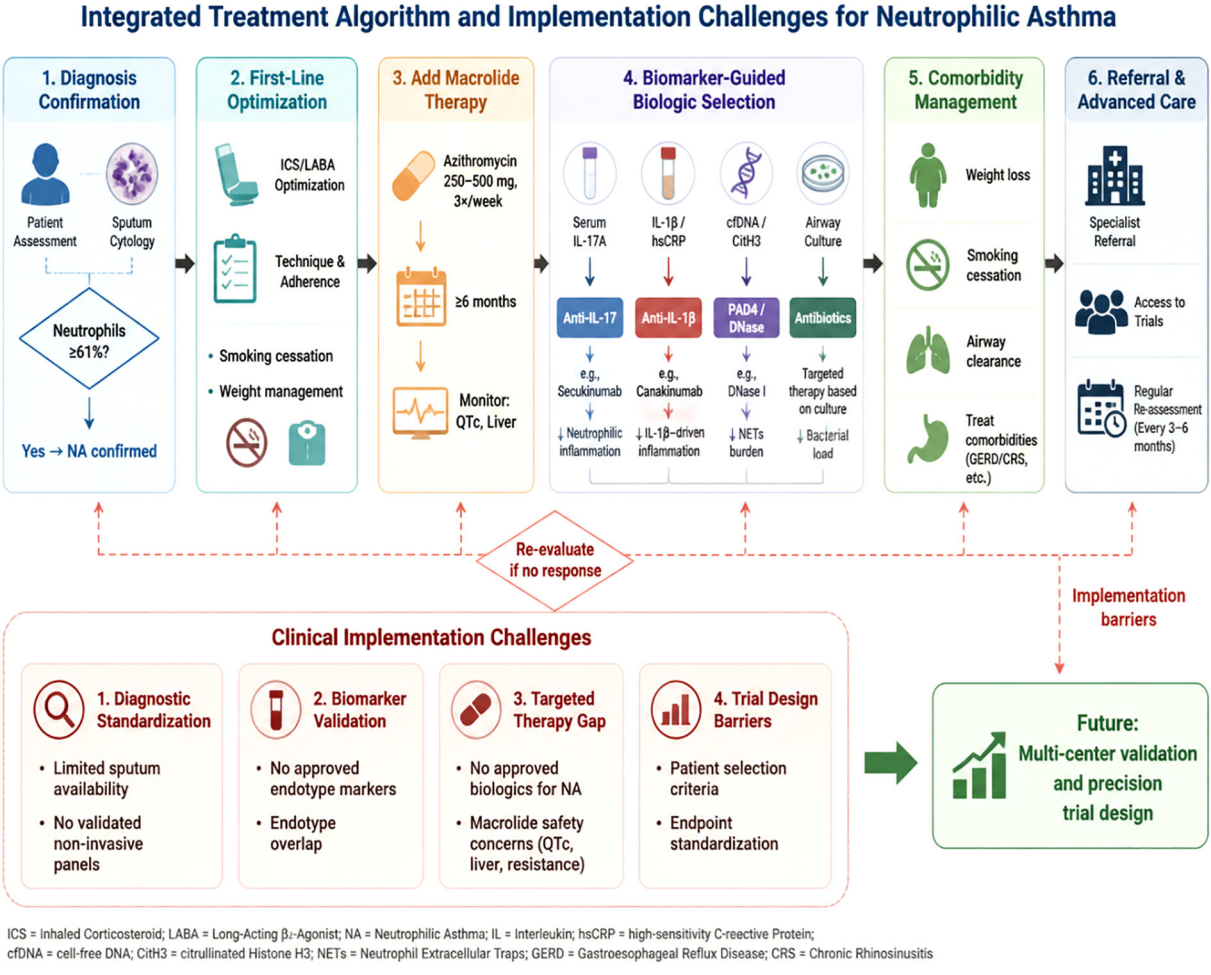
Integrated treatment algorithm and implementation challenges. Clinical decision pathway for neutrophilic asthma management with implementation barriers. Treatment algorithm: Step 1—confirm neutrophilic phenotype via sputum cytology (neutrophils ≥61%); Step 2—optimize ICS/LABA and address modifiable factors (inhaler technique, adherence, smoking cessation, weight management); Step 3—add maintenance macrolide (azithromycin 250–500 mg three times weekly) for persistent symptoms; Step 4—biomarker-guided biologic selection: anti-IL-17 (secukinumab) for elevated IL-17, anti-IL-1β (canakinumab) for inflammasome activation, PAD4 inhibitors/DNase for NET-associated, targeted antibiotics for infection-associated; Step 5—comorbidity management (obesity, bronchiectasis, GERD); Step 6—referral to severe asthma center for clinical trials and advanced therapies. Implementation challenges (highlighted in bottom panel): (1) diagnostic standardization—limited sputum induction availability, lack of validated non-invasive biomarker panels; (2) biomarker validation—no approved endotype-specific markers, endotype overlap in clinical practice; (3) therapeutic gaps—no approved targeted biologics for pure neutrophilic asthma, macrolide safety concerns (QTc prolongation, antimicrobial resistance); (4) clinical trial design—need for endotype-specific patient selection criteria, standardized outcome measures. Future directions require multi-center biomarker validation studies and precision trial design integrating molecular endotyping.

The identification of neutrophilic asthma as a distinct phenotype with specific molecular endotypes has profound implications for clinical management. This section translates the molecular understanding and biomarker framework into practical treatment algorithms (**Figure 4**).

#### 4.1.1 Glucocorticoid optimization strategies

For patients with neutrophilic asthma demonstrating partial GC responsiveness, several optimization strategies merit consideration:

- Higher-dose inhaled corticosteroids may overcome relative resistance in some patients
- Systemic corticosteroids remain options for severe exacerbations but are not preferred for maintenance
- Addition of steroid-sparing agents (macrolide antibiotics, biologics) should be prioritized
- Monitoring for GC-related adverse effects is essential given higher cumulative doses

#### 4.1.2 Macrolide antibiotic therapy

Macrolide antibiotics, particularly azithromycin, exert immunomodulatory effects beyond antimicrobial activity (38, 45). Long-term low-dose azithromycin (250–500 mg three times weekly) reduces exacerbation frequency in severe asthma, with potential enhanced benefit in neutrophilic phenotypes (44, 45). Proposed mechanisms include inhibition of neutrophil chemotaxis, reduction of IL-8 production, and impairment of bacterial quorum sensing (38, 45). Clinical trials demonstrate 30-40% reduction in exacerbations in patients with neutrophilic asthma receiving maintenance azithromycin (44, 45).

#### 4.1.3 Targeted biologic selection

Current and emerging biologics require careful patient selection based on inflammatory phenotype (30, 46):

- Anti-IL-5 agents (mepolizumab, benralizumab): Not preferred for pure neutrophilic asthma; reserved for overlap phenotypes with eosinophilia (30, 47)
- Anti-IL-4Rα (dupilumab): Limited data in neutrophilic asthma; may benefit patients with mixed inflammatory patterns (30)
- Anti-IL-17 agents (secukinumab, ixekizumab): Emerging data suggest benefit in IL-17-dominant endotype; clinical trials ongoing (11, 18)
- Anti-IL-1β (canakinumab) and IL-1 receptor antagonist (anakinra): Potential role in inflammasome-activated endotype; requires biomarker-guided patient selection (15, 30)
- Anti-TSLP (tezepelumab): May benefit broad inflammatory phenotypes; effects on neutrophilic inflammation under investigation (46, 48).
- Detailed mechanisms of action, development stages, and key clinical findings for all emerging agents are provided in **Supplementary Table 2**.

Antimicrobial Interventions: For infection-associated neutrophilic asthma:

- Targeted antibiotic therapy based on airway culture and sensitivity
- Consider chronic suppressive therapy (azithromycin) for persistent bacterial colonization
- Evaluate for bronchiectasis and refer for comprehensive airway clearance program
- Microbiome-modulating interventions (probiotics, prebiotics) remain investigational

Non-Pharmacological Approaches:

- Smoking cessation is essential for current smokers with neutrophilic asthma
- Weight management in obese patients may reduce systemic inflammation
- Exercise training improves symptoms and quality of life without altering inflammatory phenotype
- Airway clearance techniques for patients with chronic sputum production

Treatment Escalation Algorithm for Neutrophilic Asthma:

1. Confirm neutrophilic phenotype through sputum cytology
2. Optimize inhaled therapy (ICS/LABA combination)
3. If persistent symptoms/exacerbations: Add azithromycin 250–500 mg three times weekly
4. If persistent: Consider biomarker-guided biologic selection (anti-IL-17, anti-IL-1β)
5. Address comorbidities: Weight management, smoking cessation, infection treatment
6. Referral to severe asthma center for phenotyping and access to clinical trials

### 4.2 IL-17 pathway inhibitors

Targeting the IL-17 pathway addresses the central driver of neutrophil recruitment in neutrophilic asthma.

#### 4.2.1 Secukinumab

A human monoclonal antibody against IL-17A, approved for psoriasis, psoriatic arthritis, and ankylosing spondylitis (30). Early-phase trials in asthma show reduction in sputum neutrophils and improved symptom scores in patients with elevated IL-17 (18, 30). Ongoing phase 2/3 trials are evaluating efficacy in neutrophilic asthma specifically (11, 30).

#### 4.2.2 Ixekizumab

Another anti-IL-17A monoclonal antibody with similar indications. Limited data in asthma but mechanistic rationale for benefit in IL-17-dominant endotype.

#### 4.2.3 Brodalumab

An anti-IL-17 receptor A (IL-17RA) antibody blocking signaling of multiple IL-17 family cytokines. Phase 2 trials in asthma showed numerical improvement in neutrophilic subgroups but did not meet primary endpoints. Development in asthma discontinued.

#### 4.2.4 Challenges and considerations

IL-17 pathway inhibition carries risk of mucocutaneous candidiasis due to impaired neutrophil function at barrier surfaces. Patients require monitoring for opportunistic infections. Additionally, IL-17 pathway blockade may not address upstream inflammasome activation or downstream NET formation, potentially limiting efficacy as monotherapy.

Full clinical development status for IL-17 pathway inhibitors is summarized in **Supplementary Table 2**.

### 4.3 NLRP3 inflammasome inhibitors

Direct targeting of NLRP3 inflammasome represents a promising therapeutic approach, with several agents in clinical development.

#### 4.3.1 MCC950/CRID3

A small-molecule NLRP3 inhibitor that blocks ATP-induced conformational changes required for inflammasome assembly. Preclinical studies demonstrate reduction in airway inflammation and hyperresponsiveness in murine asthma models. Currently in phase 2 trials for inflammatory conditions.

#### 4.3.2 Dapansutrile (OLT1177)

An oral NLRP3 inhibitor in clinical development. Phase 2 trials in heart failure show favorable safety profile. Early-phase trials in respiratory disease are anticipated.

#### 4.3.3 Canakinumab

An anti-IL-1β monoclonal antibody approved for cryopyrin-associated periodic syndromes (15, 30). *Post-hoc* analysis of CANTOS cardiovascular trial demonstrated reduced asthma incidence, suggesting potential benefit (15). Biomarker-guided patient selection (elevated IL-1β or hsCRP) may identify responders (15, 27).

#### 4.3.4 Anakinra

An IL-1 receptor antagonist blocking both IL-1α and IL-1β. Small studies in severe asthma show reduction in sputum neutrophils in selected patients. Short half-life requires daily injection, limiting practicality.

Additional NLRP3 and IL-1 pathway inhibitors in clinical development are listed in **Supplementary Table 2**.

### 4.4 NET-targeted therapies

Therapeutic targeting of NET formation or enhancing NET clearance represents an emerging approach for NET-associated neutrophilic asthma.

#### 4.4.1 PAD4 inhibitors

Peptidylarginine deiminase 4 (PAD4) mediates histone citrullination required for chromatin decondensation during NETosis (12, 24). Small-molecule PAD4 inhibitors (e.g., GSK484, BB-Cl-amidine) reduce NET formation in preclinical models (24). Clinical development for neutrophilic diseases is ongoing (12, 26).Clinical development status for NET-targeted agents is detailed in **Supplementary Table 2**.

#### 4.4.2 DNase therapies

Recombinant human DNase I (dornase alfa) degrades extracellular DNA, including NET scaffolds. While approved for cystic fibrosis, DNase therapy in asthma remains investigational. Theoretical benefits include reduced mucus viscosity and impaired NET-mediated inflammation.

#### 4.4.3 Neutrophil elastase inhibitors

Neutrophil elastase, released during NET formation, contributes to tissue damage and mucus hypersecretion. Elastase inhibitors (e.g., AZD9668) have been evaluated in bronchiectasis and COPD with limited efficacy. Development in neutrophilic asthma has not been pursued.

### 4.5 Microbiome-targeted interventions

Modulating the airway or g**ut microbiome to restore eubiosis** represents a novel therapeutic frontier.

#### 4.5.1 Probiotics and prebiotics

Systematic reviews of probiotics in asthma show modest benefits in symptom control without clear effects on inflammation. However, strain-specific effects and targeted delivery to the airway have not been adequately studied. Preclinical data support specific strains (Lactobacillus rhamnosus GG, Bifidobacterium breve) for immunomodulatory effects.

#### 4.5.2 Fecal microbiome transplantation

Case reports and small series suggest benefit of FMT in severe asthma, though controlled trials are lacking. Mechanistic rationale includes restoration of SCFA-producing gut bacteria and reduction of systemic inflammation.

#### 4.5.3 Targeted antibiotics

Culture-directed antibiotic therapy for chronic airway infection reduces bacterial load and associated inflammation. Azithromycin, beyond immunomodulatory effects, targets common airway pathogens. Novel narrow-spectrum antibiotics and bacteriophage therapy offer potential for precision antimicrobial approaches.

### 4.6 Combination and future therapies

Given the interconnected pathogenic network in neutrophilic asthma, combination therapy targeting multiple pathways may provide enhanced benefit.

#### 4.6.1 Dual IL-17 and NLRP3 inhibition

Preclinical evidence suggests additive effects of combined IL-17 and inflammasome inhibition. Clinical development of combination approaches is anticipated.

#### 4.6.2 Triple therapy approaches

Integration of immunomodulatory (azithromycin), biologic (anti-IL-17 or anti-IL-1β), and anti-inflammatory (optimized inhaled therapy) approaches may maximize outcomes. Personalized treatment algorithms based on endotype profiling will guide combination selection.

#### 4.6.3 Emerging targets

Novel targets under investigation include:

- CXCR2 antagonists: Block neutrophil chemotaxis (danirixin, navarixin)
- G-CSF receptor antagonists: Reduce neutrophil production and survival
- Sphingosine-1-phosphate (S1P) modulators: Affect lymphocyte trafficking
- JAK inhibitors: Target cytokine signaling pathways
- Epigenetic modulators: Target HDAC dysfunction in GC resistance

## 5 Discussion

This review synthesizes current understanding of neutrophilic asthma pathogenesis and presents a precision medicine framework for diagnosis and treatment (38, 50). The molecular mechanisms—Th17/IL-17 axis, NET formation, NLRP3 inflammasome activation, and microbiome dysbiosis—form an interconnected network perpetuating neutrophilic inflammation and glucocorticoid resistance (21, 50). This complexity explains the heterogeneous treatment responses observed clinically and underscores the need for multi-targeted therapeutic approaches (38, 49).

The precision identification framework addresses a critical clinical need: distinguishing neutrophilic asthma patients who will not benefit from conventional glucocorticoid therapy and guiding selection of alternative treatments (35, 36). Integration of molecular endotyping with clinical phenotyping enables tailored therapeutic approaches (38, 49). However, challenges remain in translating this framework to clinical practice (38, 45). Sputum induction, while the gold standard for inflammatory phenotyping, requires specialized expertise and is not universally available (4, 44). Peripheral blood biomarkers lack sufficient accuracy for definitive diagnosis (37, 38). Development of non-invasive, accurate biomarker panels and point-of-care testing remains a research priority (38, 45).

The therapeutic landscape for neutrophilic asthma is evolving rapidly (40, 43). While no targeted therapies are currently approved specifically for neutrophilic asthma, several candidates show promise (19, 43). IL-17 pathway inhibitors, NLRP3 inflammasome inhibitors, and NET-targeted therapies are in various stages of clinical development (8, 15, 19). The challenge lies in matching the right therapy to the right patient—a precision medicine approach requiring validated biomarkers and endotype classification systems (35, 38, 45).

Limitations of this review should be acknowledged. The molecular mechanisms discussed are based on evolving science, with some findings derived from preclinical models requiring human validation. The precision identification framework represents a proposed approach that has not been prospectively validated. Therapeutic recommendations, while evidence-based where possible, incorporate expert opinion given the limited approved options for neutrophilic asthma.

## 6 Future perspectives

The field of neutrophilic asthma research is advancing rapidly, with several areas poised for breakthrough developments:

### 6.1 Single-cell multi-omics

Application of single-cell RNA sequencing, proteomics, and epigenomics to airway samples will enable unprecedented resolution of cellular heterogeneity and endotype classification. Integration with clinical outcomes will refine precision medicine approaches.

### 6.2 Artificial intelligence for endotyping

Machine learning algorithms applied to multi-omics data, clinical characteristics, and treatment outcomes will enable data-driven endotype discovery and predictive modeling for treatment response.

### 6.3 Novel drug delivery systems

Targeted delivery of therapeutics to airway neutrophils or specific cell populations may enhance efficacy while minimizing systemic effects. Nanoparticle-based delivery and inhaled biologics represent active areas of development.

### 6.4 Microbiome engineering

Beyond probiotics, next-generation microbiome interventions including engineered bacteria, phage therapy, and postbiotic metabolites offer potential for precise microbiome modulation.

### 6.5 Regenerative medicine

Airway epithelial repair and restoration of barrier function represent emerging therapeutic targets. Stem cell-derived therapies and growth factor delivery may complement anti-inflammatory approaches.

### 6.6 Preventive strategies

Understanding early-life determinants of neutrophilic asthma (microbiome establishment, viral infections, environmental exposures) may enable primary prevention strategies in at-risk populations.

## 7 Conclusions

Neutrophilic asthma represents a distinct inflammatory phenotype characterized by glucocorticoid resistance and severe disease course (4, 50). The molecular mechanisms—Th17/IL-17 axis, NET formation, NLRP3 inflammasome activation, and microbiome dysbiosis—create self-reinforcing inflammatory loops requiring targeted therapeutic approaches (7, 19, 50). The precision identification framework integrating molecular endotypes, biomarker profiles, and clinical phenotypes provides a roadmap for personalized treatment (35, 38). Emerging therapies targeting IL-17, NLRP3 inflammasome, and NET formation, combined with microbiome-modulating interventions, offer promise for improved outcomes in this difficult-to-treat asthma phenotype (7, 15, 19, 33). Translating these advances to clinical practice requires validated biomarkers, prospective clinical trials, and integration of precision medicine approaches into routine asthma care (36, 48).

## References

[1] Simpson JL Scott R Boyle MJ Gibson PG . Inflammatory subtypes in asthma: assessment and definition using induced sputum. Respirology. (2006) 11:54–61. doi: 10.1111/j.1440-1843.2006.00783.x 16423202

[2] Woodruff PG Modrek B Choy DF Jia G Abbas AR Ellsworth A . T-helper type 2-driven inflammation defines major subphenotypes of asthma. Am J Respir Crit Care Med. (2009) 180:388–95. doi: 10.1164/rccm.200903-0392OC PMC2742757 19483109

[3] Fahy JV . Type 2 inflammation in asthma — present in most, absent in many. Nat Rev Immunol. (2015) 15:57–65. doi: 10.1038/nri3786 25534623 PMC4390063

[4] Moore WC Hastie AT Li X Li H Busse WW Ampleford EJ . Sputum neutrophil counts are associated with more severe asthma phenotypes using cluster analysis. J Allergy Clin Immunol. (2014) 133:1557–63. doi: 10.1016/j.jaci.2013.10.011 24332216 PMC4040309

[5] Haldar P Pavord ID Shaw DE Berry MA Thomas M Brightling CE . Cluster analysis and clinical asthma phenotypes. Am J Respir Crit Care Med. (2008) 178:218–24. doi: 10.1164/rccm.200711-1754OC 18480428 PMC3992366

[6] Barnes PJ . Mechanisms of glucocorticoid resistance in severe asthma. Allergy. (2023) 78:420–32. doi: 10.1111/all.15585 36385701

[7] Hastie AT Moore WC Meyers DA Vestal PL Li H Peters SP . Analyses of neutrophilic vs eosinophilic severe asthma. Am J Respir Crit Care Med. (2017) 195:1619–28. doi: 10.1016/j.jaci.2010.02.008

[8] Fahy JV Kim RY Simpson JL Liu J Fulbridge L Lee J . Neutrophilic asthma: phenotypes and mechanisms. Allergy. (2022) 77:3006–19. doi: 10.1111/all.15366 35538848

[9] Barnes PJ Adcock IM . Glucocorticoid resistance in inflammatory diseases. Lancet. (2009) 373:1905–17. doi: 10.1016/S0140-6736(09)60326-3 19482216

[10] Newcomb DC Peebles RS . Th17-mediated inflammation in asthma. Curr Opin Immunol. (2013) 25:755–60. doi: 10.1016/j.coi.2013.09.002 24035139 PMC3855890

[11] Chaudhary N McAuley J El Gamal A Tay J Hsu AC Foster PS . The role of the IL-17/IL-23 axis in neutrophilic asthma. Allergy. (2022) 77:463–71. doi: 10.1111/all.15123 34608654

[12] Brinkmann V Reichard U Goosmann C Fauler B Uhlemann Y Weiss DS . Neutrophil extracellular traps kill bacteria. Science. (2004) 303:1532–5. doi: 10.1126/science.1092385 15001782

[13] Williams EJ Wood LG Dowling LRC Stanton S Baines KJ . Neutrophil extracellular traps are increased in severe asthma. Am J Respir Crit Care Med. (2021) 204:464–72. doi: 10.1183/23120541.00025-2025

[14] Schroder K Tschopp J . The inflammasomes. Cell. (2010) 140:821–32. doi: 10.1016/j.cell.2010.01.040 20303873

[15] Standiford TJ Kuick R Bhan U Christensen PJ Danasty K Evanoff H . NLRP3 inflammasome activation by Th17-derived cytokines in severe asthma. Am J Respir Crit Care Med. (2019) 199:1262–74. doi: 10.1164/rccm.201805-0941OC

[16] Huang YJ Boushey HA . The microbiome in asthma. J Allergy Clin Immunol. (2015) 135:25–30. doi: 10.1016/j.jaci.2014.11.028 25567040 PMC4287960

[17] Trompette A Gollwitzer ES Yadava K Sichelstiel AK Sprenger N Ngom-Bru C . Gut microbiota influences airway inflammation via metabolites. Nat Med. (2014) 20:159–66. doi: 10.1038/nm.3444 24390308

[18] Al-Ramli W Préfontaine D Chouiali F Kim JM Fiorentino C Ramasamy J . TH17-associated cytokines (IL-17A and IL-17F) in severe asthma. J Allergy Clin Immunol. (2009) 123:1185–7. doi: 10.1016/j.jaci.2009.02.024 19361847

[19] Wei Q Liao J Jiang M Liu J Liang X Nong G . Relationship between Th17-mediated immunity and airway inflammation in childhood neutrophilic asthma. Allergy, Asthma & Clinical Immunology. (2021) 17(1):4. doi: 10.1186/s13223-020-00504-3 PMC7789788 33407843

[20] Wakashin H Hirose K Maezawa Y Kagami SI Watanabe N Suto A . IL-23 and Th17 cells enhance Th2 cell-mediated eosinophilic airway inflammation in mice. Am J Respir Crit Care Med. (2008) 178:1023–32. doi: 10.1164/rccm.200801-128OC 18787221

[21] Cosmi L Maggi L Santarlasci V Liotta F Frosali D Angeli R . Identification of a novel subset of human circulating memory CD4+ T cells that produce both IL-17A and IL-4. J Allergy Clin Immunol. (2010) 125:222–30. doi: 10.1016/j.jaci.2009.10.019 20109749

[22] van Beveren GJ Said H van Houten MA Bogaert D . The respiratory microbiome in childhood asthma. J Allergy Clin Immunol. (2023) Dec; 152(6):1352–1367. doi: 10.1016/j.jaci.2023.10.001 37838221

[23] Segal LN Clemente JC Tsay JC Wu BG Wang Y Watkins T . Enrichment of the lung microbiome with oral commensals in severe asthma. Am J Respir Crit Care Med. (2019) 199:1250–61. doi: 10.1164/rccm.201807-1605OC

[24] Silvestre-Roig C Brinkmann V Glogauer M . Neutrophil extracellular traps in airway inflammation. Cell Tissue Res. (2021) 385:495–507. doi: 10.1007/s00441-021-03450-2 30311153

[25] Dworski R Simon J Shopp J He R Hawkins G Han W . Tissue-specific neutrophil extracellular traps in asthma. J Allergy Clin Immunol. (2022) 149:956–67. doi: 10.1016/j.jaci.2021.11.003 34798040

[26] Li Y Liu J Hu Y Cong CZ Chen YM Zhou Q . Crossroads: Pathogenic role and therapeutic targets of neutrophil extracellular traps in rheumatoid arthritis. BIOCELL. (2024) 48(1). doi: 10.32604/biocell.2023.045862

[27] De Volder J Vereecke L Joos G Maes T . Targeting neutrophils in asthma: A therapeutic opportunity?. Biochem Pharmacol. (2020) 182:114292. doi: 10.1016/j.bcp.2020.114292 33080186

[28] Horvat JC Kim RY Weaver N Augood C Brown AC Donovan C . Characterization and inhibition of inflammasome responses in severe and non-severe asthma. Respir Res. (2023) 24(1):303. doi: 10.1186/s12931-023-02603-2 38044426 PMC10694870

[29] Goossens J Jonckheere AC De Boodt S Dilissen E Marain N Decaesteker T . Sputum Transcriptomic Analysis and Clustering Reveals Insight Into Asthma Heterogeneity. Lung. (2025) 203(1):89. doi: 10.1007/s00408-025-00843-1 40830242

[30] Busse WW . Biological treatments for severe asthma: a comprehensive review. J Allergy Clin Immunol. (2019) 143:22–32. doi: 10.1016/j.jaci.2018.09.018 30312705

[31] Quek E Horn N Siddiqui S . Precision Medicine in Asthma: The Role of Biomarkers. Immunotherapy Targets and Therapy. (2025) 14:1479–1513. doi: 10.2147/ITT.S532291 PMC12756974 41488813

[32] Kaur D Hollins F Saunders R Woodman L Addis G Amrani Y . Neutrophil apoptosis is delayed in severe asthma: role of Mcl-1 and GM-CSF. J Allergy Clin Immunol. (2020) 145:1570–80. doi: 10.1016/j.jaci.2019.12.911 31953105 PMC7282965

[33] Ouyang L Su G Quan J Xiong Z Lai T . Emerging roles and therapeutic implications of HDAC2 and IL-17A in steroid-resistant asthma. Chin Med J Pulmonary Crit Care Med. (2023) 1(2):108–112. doi: 10.1016/j.pccm.2023.04.003 39170824 PMC11332885

[34] Bi J Min Z Yuan H Jiang Z Mao R Zhu T . PI3K inhibitor treatment ameliorates the glucocorticoid insensitivity of PBMCs in severe asthma. Clin Transl Med. (2020) 9(1):22. doi: 10.1186/s40169-020-0262-5 32112175 PMC7048898

[35] Hildebrand CB Lichatz R Pich A Mühlfeld C Woltemate S Vital M . Short-chain fatty acids improve inflamm-aging and acute lung injury in old mice. Am J Physiol Lung Cell Mol Physiol. (2023) 324(4):L480–L492. doi: 10.1152/ajplung.00296.2022 36802219

[36] Hilty M Burke C Pedro H Cardenas P Cooper A Huffnagle GB . Disordered microbial communities in asthmatic airways. PloS One. (2010) 5:e8578. doi: 10.1371/journal.pone.0008578 20052417 PMC2798952

[37] Arrieta MC Stiemsma LT Dimitriu PA Wong J Russell T Johnson C . Early infancy microbial and metabolic alterations affect risk of childhood asthma. Sci Transl Med. (2015) 7:275ra22. doi: 10.1126/scitransmed.aaa0073 26424567

[38] Taylor SL Leong LEX Mobegi FM Choo JM Wesselingh S . Long-term azithromycin in severe asthma reduces airway microbiome diversity. Eur Respir J. (2019) 54:1900285. doi: 10.1164/rccm.201809-1739OC 40074277

[39] Kaur R Chupp G . Phenotypes and endotypes of adult asthma: Moving toward precision medicine. J Allergy Clin Immunol. (2019) 144(1):1–12. doi: 10.1016/j.jaci.2019.05.031 31277742

[40] International ERS/ATS guidelines on definition, evaluation and treatment of severe asthma Chung KF Wenzel SE Brozek JL . International ERS/ATS guidelines on definition, evaluation and treatment of severe asthma. Eur Respir J. (2014) 43:343–73. doi: 10.1183/09031936.00202013 24337046

[41] Fajt ML Wenzel SE . Asthma phenotypes and endotypes: implications for personalized medicine. J Allergy Clin Immunol Pract. (2021) 9:61–8. doi: 10.1016/j.jaip.2020.09.035 33429705

[42] Brinkman P Wagener AH Hekking PP Bansal AT Maitland-van der Zee AH Wang Y . Identification and prospective stability of electronic nose (eNose)-derived inflammatory phenotypes in patients with severe asthma. J Allergy Clin Immunol. (2019) 143(5):1811–1820.e7. doi: 10.1016/j.jaci.2018.10.058 30529449

[43] Wenzel SE . Asthma phenotypes: the evolution from clinical to molecular approaches. Nat Med. (2012) 18:716–25. doi: 10.1038/nm.2678 22561835

[44] McDonald VM Higgins I Wood LG Gibson PG . Multidimensional assessment and tailored interventions for severe asthma: a comprehensive approach. Respirology. (2013) 18:622–31. doi: 10.1111/resp.12066 23418922

[45] Gibson PG Yang IA Upham JW Baines KJ Simpson JL Reynolds JN . Effect of azithromycin on asthma exacerbations and quality of life in adults with persistent uncontrolled asthma (AMAZES): a randomised double-blind placebo-controlled trial. Lancet. (2017) 390:659–68. doi: 10.1016/S0140-6736(17)31281-3 28687413

[46] Menzies-Gow A Corren J Bourdin A Chupp G Israel E Wechsler ME . Tezepelumab in Adults and Adolescents with Severe, Uncontrolled Asthma. N Eng J Med. (2021) 384(19):1800–1809. doi: 10.1056/NEJMoa2034975 33979488

[47] Haldar P Brightling CE Hargadon B Gupta S Monteiro W Sousa A . Mepolizumab and exacerbations of refractory eosinophilic asthma: a proof-of-concept study. Lancet. (2009) 374:652–9. doi: 10.1056/NEJMoa0808991 19264686 PMC3992367

[48] Corren J Parnes JR Wang L Mo M Roseti SL Griffiths JM . Tezepelumab reduces exacerbations in severe, uncontrolled asthma. J Allergy Clin Immunol. (2021) 148:1438–49. doi: 10.1016/j.jaci.2021.08.019 34492259

[49] Gibeon G Baines KJ O'Grady N O'Connor BJ Williams M Williams P . Fatigue in severe asthma: relationships with disease phenotype and clinical outcomes. Respirology. (2019) 24:1021–8. doi: 10.1111/resp.13598 40046247

[50] Global Initiative for Asthma . Global strategy for asthma management and prevention (2024). Available online at: https://ginasthma.org (Accessed June 12, 2026).

