# Supplementary material for: Precision identification and targeted therapy for neutrophilic asthma: from molecular mechanisms to clinical translation
Source: Front Immunol. 2026 Jul 8;17:1878339. doi: 10.3389/fimmu.2026.1878339 (PMC13388270; doi:10.3389/fimmu.2026.1878339)
Supplement: Supplementary file 1 [file DataSheet1.pdf]

Table S2. Emerging Therapeutic Targets and Clinical Development Status

Target	Drug Name	Mechanism of Action	Development Stage	Key Findings
**IL-17A**	Secukinumab	Human IgG1 anti-IL-17A mAb	Phase 2 (asthma)	Reduced sputum neutrophils; improved symptoms in IL-17-high subgroup
**IL-17A**	Ixekizumab	Humanized anti-IL-17A mAb	Phase 2 (asthma)	Ongoing; mechanistic rationale established
**IL-17RA**	Brodalumab	Anti-IL-17RA mAb	Discontinued (asthma)	Numerical improvement in neutrophilic subgroup; primary endpoint not met
**IL-1β**	Canakinumab	Human anti-IL-1β mAb	Phase 2 (respiratory)	Reduced asthma incidence in CANTOS trial; biomarker selection needed
**IL-1R**	Anakinra	IL-1 receptor antagonist	Phase 2 (severe asthma)	Reduced sputum neutrophils in selected patients; short half-life limits utility
**NLRP3**	MCC950	Small molecule NLRP3 inhibitor	Phase 2 (inflammatory)	Effective in preclinical asthma models; human trials anticipated
**NLRP3**	Dapansutrile	Oral NLRP3 inhibitor	Phase 2 (cardiovascular)	Favorable safety; respiratory trials planned
**PAD4**	BB-Cl-amidine	Pan-PAD inhibitor	Preclinical	Reduces NET formation; in vivo data limited
**CXCR2**	Danirixin	CXCR2 antagonist	Phase 2 (COPD/asthma)	Reduced sputum neutrophils; clinical efficacy unclear
**CXCR2**	Navarixin	CXCR2 antagonist	Phase 2 (asthma)	Reduced neutrophil recruitment; development paused

Abbreviations: COPD, chronic obstructive pulmonary disease; CXCR2, C-X-C chemokine receptor 2; IgG1, immunoglobulin G1; IL, interleukin; mAb, monoclonal antibody; NLRP3, NOD-like receptor protein 3; PAD4, peptidylarginine deiminase 4.
